# Numerical evaluation of face masks for prevention of COVID-19 airborne transmission

**DOI:** 10.1007/s11356-022-18587-3

**Published:** 2022-02-09

**Authors:** Jiaxing Liu, Ming Hao, Shulei Chen, Yang Yang, Jian Li, Qi Mei, Xin Bian, Kun Liu

**Affiliations:** 1 School of Mechanical Engineering and Automation, Northeastern University, Shenyang, Liaoning People’s Republic of China; 2 Department of Ophthalmology, Renmin Hospital of Wuhan University, Wuhan, Hubei People’s Republic of China; 3 Institute of Experimental Immunology, University Clinics of Rheinische Friedrich-Wilhelms-University, Bonn, Germany; 4 Department of Oncology, Tongji Hospital, Tongji Medical College, Huazhong University of Science and Technology, Wuhan, Hubei People’s Republic of China; 5 School of Aeronautics and Astronautics, Zhejiang University, Hangzhou, Zhejiang People’s Republic of China

**Keywords:** COVID-19, Face masks, Computational fluid dynamics, Cough droplet lifetime, Exposure risks, Ventilated rooms

## Abstract

The COVID-19 pandemic has forced governments around the globe to apply various preventive measures for public health. One of the most effective measures is wearing face masks, which plays a vital role in blocking the transmission of droplets and aerosols. To understand the protective mechanism of face masks, especially in indoor environments, we apply a computational fluid dynamics technique to predict the lifetime of cough droplets. Therefore, we can assess the exposure risk in a ventilated room where an infected individual wears a face mask or not. We focus on the dynamic evaporation and diffusion of droplets in a human-cough process, which is a major cause for the spread of the virus. We find that wearing a face mask can effectively reduce the total mass and Sauter mean diameter of the residual droplets after a single cough. The mass concentration of virus-carrying droplets in the ventilated room decreases by 201, 43,786, and 307,060 times, corresponding to wearing cotton face masks, surgical face masks, and N95 face masks, respectively. However, the maximum travel distance of 80% droplets is insensitive to wearing a face mask or not. Therefore, the residual droplets are widely distributed due to the influence of indoor airflow. Furthermore, we study aerosol exposure risks in different areas of the room and find that high concentrations of aerosols occur in the streamline through an infected individual, especially next to the individual within 1.5 m. This strongly suggests a social distance despite the fact that the majority of droplets are filtered by face masks. This study explains the impact of face masks and airflow on indoor exposure risks and further inspires potential measures for public health, for example, no individuals should sit near the air supply opening.

## Introduction

There is a concerning development that coronavirus disease 2019 (COVID-19) cases continue to rebound globally (O’Dowd 2021). One of the reasons is the rapid spread of delta variant, which is declared as a “variant of concern” by the Centers for Disease Control and Prevention (CDC) (Kupferschmidt and Wadman 2021). Various measures have been adopted to alleviate, control, and stop the spread of COVID-19 (Alon et al. 2020; Dhama et al. 2020; Sabat et al. 2020). Vaccination is considered to be the most effective means to control the epidemic (Christie et al. 2021). However, the effectiveness of vaccines against variants may decrease, especially for people who have only received the first dose (Farinholt et al. 2021; Lopez Bernal et al. 2021). Personal protection is necessary to avoid virus-carrying droplets. Therefore, many countries and international organizations advise people to wear face masks in public even though they have been vaccinated (Liao et al. 2021).

The effectiveness and potential impact of face masks have been intensely discussed. Leung et al. (2020) conducted a statistical analysis including 3,363 individuals. Their results show that there is a significant drop in the rate of influenza virus residing in the respiratory system for individuals with face masks. Eikenberry et al. (2020) developed a modified SEIR epidemic model to assess the impact of face masks, which is driven by partial differential equations. Their study demonstrated that the use of face masks helps control the COVID-19 pandemic. Meanwhile, the implementation of wearing face masks has also caused some concerns. For instance, wearing face masks may bring individuals a false sense of security, thereby causing a higher risk of virus transmission (Yan et al. 2021). In addition, extensive use of face masks poses significant challenges to the terrestrial and marine environment (Selvaranjan et al. 2021). Sustainable pollution management measures should be considered to tackle environmental challenges (Awan 2020; Awan et al. 2020, 2021; Kanwal and Awan 2021).

The performance of face masks to prevent the transmission of SARS-CoV-2 virus depends strongly on the fluid dynamics of virus-carrying droplets. The virus can be transmitted through a variety of ways, such as direct contact with mucus, traveling of large droplets, and even suspension of aerosols (van Doremalen et al. 2020). Cough is one of the most commonly experienced symptoms of COVID-19 patients, which expels a large quantity of droplets. These droplets are considered the main media for virus transmission (Desai and Patel 2020; Zhu et al. 2020). Face masks provide a barrier to prevent the transmission of droplets and aerosols. Due to the resistance of face masks to cough airflow, the droplets and the aerosols exhibit complex dynamic behavior. To reduce the risk of infection, it is necessary to understand the fluid dynamics and the protective mechanism of face masks. Table 1 shows the recent literature that demonstrated the effectiveness of face masks to prevent the transmission of virus-carrying droplets.

**Table 1:** Literature on effectiveness of face masks

Authors	Research methods	Environmental conditions	Main results
Dbouk and Drikakis (2020)	Numerical simulation	Stagnant air condition	Most droplets travel to about 70 cm without wearing face masks while only about half the distance with face masks
Ueki et al. (2020)	Viral load measurement	Stagnant air condition	Face masks inhibit transmission of infective droplets/aerosols, but it is difficult to block viruses completely even with N95 face masks
Bandiera et al. (2020)	Viral load measurement	Stagnant air condition	Less than one in 1000 droplets leak from face masks for both speaking and coughing
Khosronejad et al. (2021)	Numerical simulation	Stagnant air condition	Face masks prevent droplets from traveling further than 0.72 m away from the mouth
Verma et al. (2020)	Flow visualization	Stagnant air condition	Non-medical face masks prevent the transmission of large droplets, despite the fact that flow leakage occurs
Ishii et al. (2021)	Flow visualization	Stagnant air condition	Exhaled airflow tends to reattach to the human body and cause aerosols to flow up with face masks
Kumar et al. (2020)	Numerical simulation	Stagnant air condition	By wearing a simple cotton face mask and keeping a strict physical distance of 2 m, the airborne transmission can be greatly reduced
Lee et al. (2021)	Numerical simulation	Unidirectional wind condition	The travel distance of droplets is about 20–25 cm with face masks
Feng et al. (2020)	Numerical simulation	Unidirectional wind condition	Even wearing a face mask loosely reduces significantly the suspension of cough droplets
Mirikar et al. (2021)	Numerical simulation	Indoor ventilation condition	Face masks significantly reduce the resident time of cough droplets indoors
Pendar and Páscoa (2020)	Numerical simulation	Indoor ventilation condition	During sneeze and cough, face masks reduce the travel distance of droplets to about one-third of that without face masks

The environmental conditions of the recent research mainly focus on the stagnant air condition. However, humans tend to be indoors most of the time. The use of face masks and indoor airflow may affect exposure risks in the room. Only a few studies have considered indoor airflow and showed the travel distance and final fate of virus-carrying droplets (such as sedimentation, suspension, and escape through outlet vents). However, these studies have not considered the diameter and mass concentration of virus-carrying droplets, which determine the transmission behavior of droplets and indoor exposure risks.

To fill the above research gaps, it is necessary to further investigate the potential influence of face masks on the trajectory of cough droplets and exposure risks indoors. Computational fluid dynamics (CFD) technique, especially the Lagrangian type, is pertinent to investigate more details of droplet transmission. In this study, we consider individuals with different types of face masks in a ventilated room. Face masks are considered porous media, and cough droplets are considered spherical particles. We perform systematically Euler–Lagrangian simulations to investigate the Sauter mean diameter, maximum travel distance of 80% droplets, and total mass of residual droplets in the room. Meanwhile, we assess the aerosol exposure risks in different areas of the room.

## Methods

**Fig. 1:**
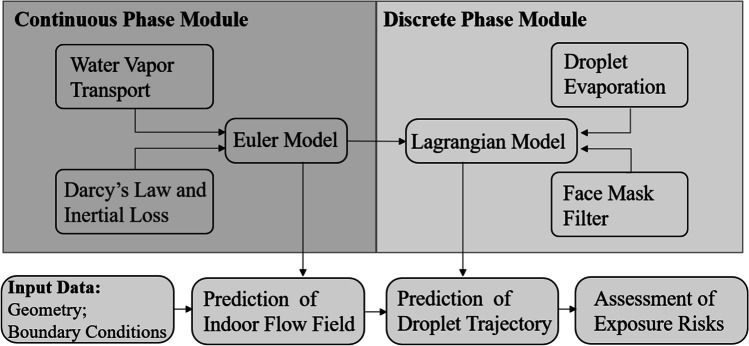
Graphical overview of simulation procedure

Exposure risks in a ventilated room with a relative humidity of 50% are studied by several numerical simulations. For different infection stages and variants, the viral load in respiratory droplets often differs by several orders of magnitude (To et al. 2020; Mahase 2021). To make the research more universal, we use aerosol concentration to quantify exposure risks. An Euler-Lagrangian model is employed to simulate indoor airflow and droplet transmission. The air is considered continuous phase, while the droplets exhaled from individuals are considered discrete phase. The simulation procedure can be roughly divided into two main modules. First, the time-dependent Euler model is used to solve indoor flow fields, and then, the Lagrangian model is used to predict the trajectory of cough droplets. In addition, the heat and mass transfer between droplets and the surrounding air is considered to predict droplet evaporation in this simulation. A graphical overview of the simulation procedure is presented in Fig. 1.

**Fig. 2:**
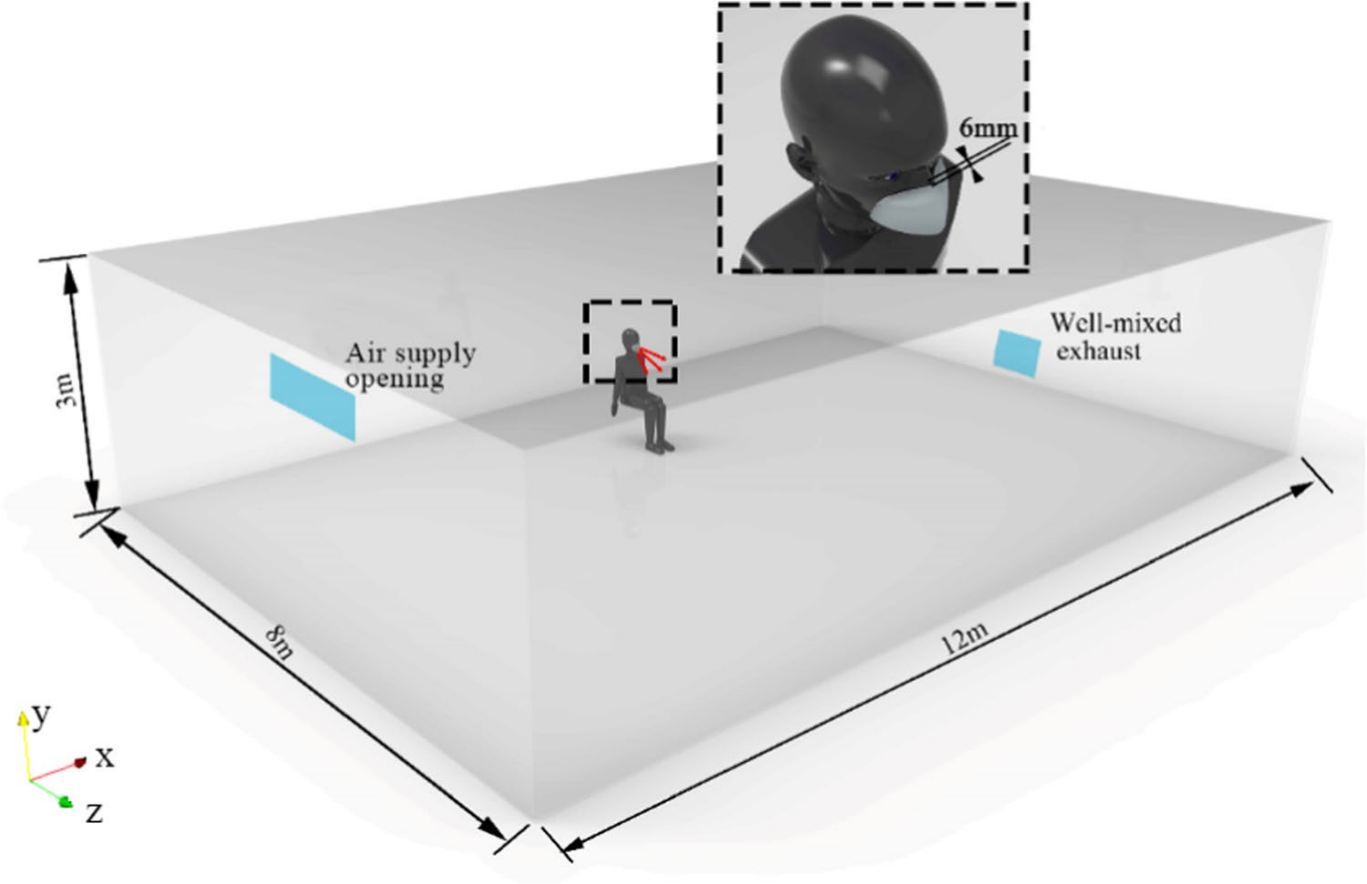
Schematic diagram of computational domain

The geometric size of the room is $12\mathrm{ m}\times 8\mathrm{ m}\times 3\mathrm{ m}$ ($\mathrm{length}\times \mathrm{width}\times \mathrm{height}$). A model of human body of 1.7 m tall and 0.6 m wide, with 310 mm^2^ mouth opening is placed in a position of *x* = 0 m, *y* = 0 m, and *z* =  − 1.5 m, as shown in Fig. 2. Unstructured hybrid polyhedron meshes and prism layers are generated for the computational domain. Five prism layers are created on the near-face region. ${y}^{+}$ is a dimensionless number to ensure the applicability of the wall function, which is defined as follows:

1 $${y}^{+}=\frac{{u}_{\tau }y}{\nu}$$

where ${u}_{\tau }$ is the shear velocity near the walls, $y$ is the distance from the wall, and $\nu$ is the kinematic viscosity of the air. The thickness of the first prism layer is 2 mm to guarantee ${y}^{+}$ < 1 (Chen 1995). The maximum skewness of the grid is 0.88. Moreover, the surfaces of face mask and human body are locally refined with the size of 1 mm and 10 mm respectively to accurately predict the airflow near the human body.

### Airflow model

Simulations are performed with Fluent 2020 R1. To verify the grid sensitivity, the computational domain is discretized into coarse grids (1.1 million cells), medium grids (2.6 million cells), and fine grids (9.4 million cells), respectively. The refinement factors and grid convergence index (GCI) corresponding to the three grids are calculated: *r*_12_ = 1.33, *r*_23_ = 1.53, *GCI*_1_ = 16.5%, and *GCI*_2_ = 3.7% for the velocity in front of the human body model (Boache 1994). *GCI*_*k*_ is defined as follows:

2 $${GCI}_{k}=F\frac{{r}_{k,k+1}^{p}{\delta }_{r(k,k+1)}}{{r}_{k,k+1}^{p}-1}$$

where *F* is the safety factor, *p* is the observed order of convergence, and ${\delta }_{r}$ is the relative error of the velocity values of the two sets of grids at the sampling point. In this study, *F* = 3, *p* = 5.22, ${\delta }_{r12}=4.26\%$, and ${\delta }_{r23}=1.11\%$. We adopt the medium grid for computation because the value of *GCI*_*2*_ is less than 5%, which is considered fine enough.

**Fig. 3:**
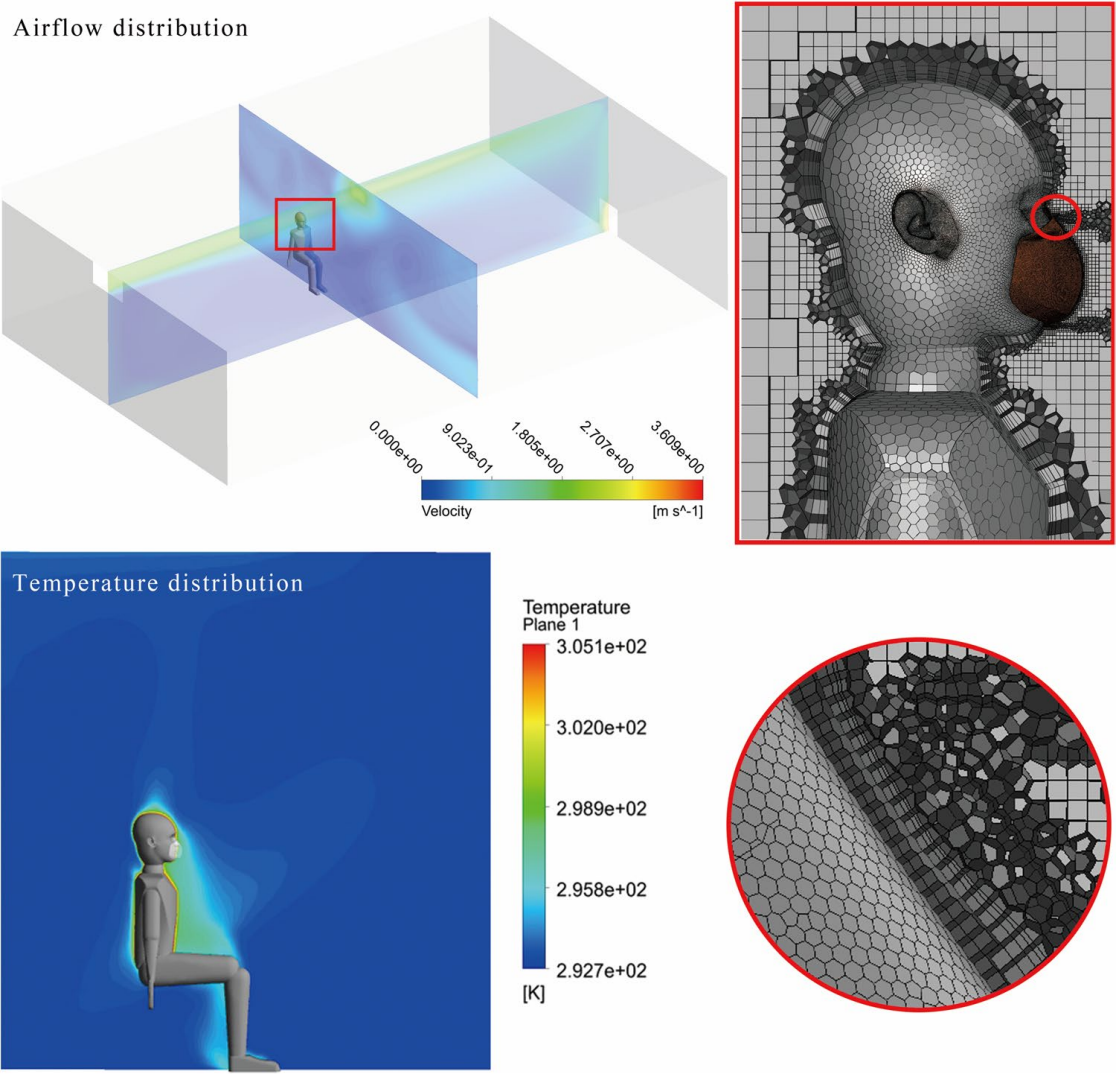
The grid details and the prediction of indoor airflow and temperature

Furthermore, we apply the re-normalization group (RNG) *k*-*ɛ* turbulence model with enhanced wall treatment in this study. The RNG model adds an additional term to the dissipation rate equation to explain the anisotropy in regions of large shear and has satisfactory performance in simulating indoor airflow (Chen 1995; Posner et al. 2003). In addition, Tao et al. compared it with other turbulence models, and their results showed that the RNG model has better performance in simulating the wake behavior around a stagnant body (Tao et al. 2017). The grid details and the prediction of indoor airflow and temperature are shown in Fig. 3. The temperature distribution in the room is approximately homogeneous, but slightly higher near the human body, which is the cause of thermal plumes.

### Cough airflow and droplets

**Fig. 4:**
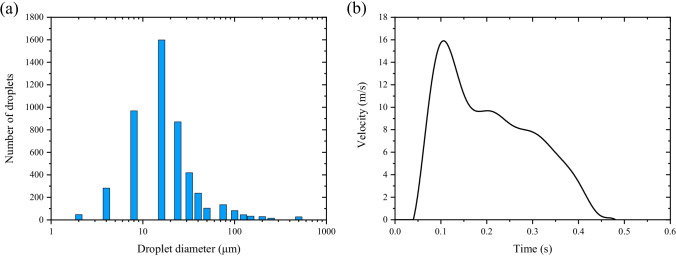
Cough-jet boundary conditions. **a** Size distribution of cough droplets. **b** Airflow velocity expelled by a single cough

Airflow and droplets expelled by coughing are investigated in this study. Duguid (1946) measured the cough droplets size distribution through a microscope, as shown in Fig. 4a. The velocity of airflow expelled by coughing is fitted by the sum of several sine functions based on experimental data of its volume flow which is measured by a spirometer based on Fleish type pneumotachograph, as shown in Fig. 4b (Gupta et al. 2009). The maximum airflow velocity is 15.9 m/s, which is similar to the study of Kwon et al.: 15.3 m/s for the males and 10.6 m/s for the females (Kwon et al. 2012).

The mass fraction of water vapor in the airflow is 5% (Li et al. 2018), and the mass transfer of water vapor is solved through the convection–diffusion equation (Theodosiu et al. 2000):

3 $$\frac{\partial(\rho Y)}{\partial t}+\operatorname{div}(\rho \vec{u} Y)=-\operatorname{div}(J)+S_{Y}$$

4 $$J=-\left({\rho D}_{m}+\frac{{\mu }_{t}}{{Sc}_{t}}\right)\mathrm{grad}(Y)$$

where $\rho$ is the density of air, $Y$ is the mass fraction of water vapor, $\stackrel{\rightharpoonup }{u}$ is the velocity of airflow, $J$ is the diffusion flux of water vapor, ${S}_{Y}$ is the rate of water vapor creation from droplets evaporation, ${D}_{m}$ is the mass diffusion coefficient for water vapor in the air mixture, ${\mu }_{t}$ is the turbulent viscosity, and $S{c}_{t}$ is the turbulent Schmidt number, which is the ratio of the kinematic viscosity coefficient to the diffusion coefficient.

### Pressure drop across face masks

A face mask is reconstructed with a maximum gap of 6 mm between the face mask and the nose, while the minimum distance is 1 mm in the side gap. The face mask is considered a porous medium, and the pressure drop across it includes two parts: Darcy’s law and inertial loss (Xi et al. 2020):

5 $$\Delta p=-\left(\frac{\nu}{a}u+\frac{1}{2}{C}_{2}{\rho u}^{2}\right)\Delta m$$

where $\nu$ is the kinematic viscosity of air, $\alpha$ is the permeability of the face mask, $u$ is the velocity normal to the face mask, ${C}_{2}$ is the inertial resistance factor, and $\Delta m$ is the thickness of the face mask. The detailed parameters of different types of face masks are shown in Table 2. In addition, the filtration efficiency for cough droplets with different diameters follows the study of Feng et al. (2020) and Shakya et al. (2017) and is shown in Table 3. A user defined function is used to generate uniform random numbers within 0 to 1 for each droplet. When the number is less than the filtration efficiency, the droplet is considered to be stuck by the face mask.

**Table 2:** Main parameters of face masks

Face mask type	Face permeability (m^2^)	Inertial resistance factor (1/m)	References
Cotton face masks	1.63 × 10^10^	94,378	Saldaeva (2009)
Surgical face masks	2.26 × 10^9^	7266	Golkarfard et al. (2019)
N95 face masks	1.12 × 10^10^	/	Zhang et al. (2016)

**Table 3:** Filter efficiency of face masks for droplets with different diameters

Face mask type	0.5 μm	1 μm	2.5 μm	4 μm	10 μm
Cotton face masks	6.2%	13.4%	22.6%	45.5%	89.3%
Surgical face masks	90.9%	98.4%	98.6%	100%	100%
N95 face masks	98.7%	99.3%	100%	100%	100%

### Droplet transport and evaporation

**Fig. 5:**
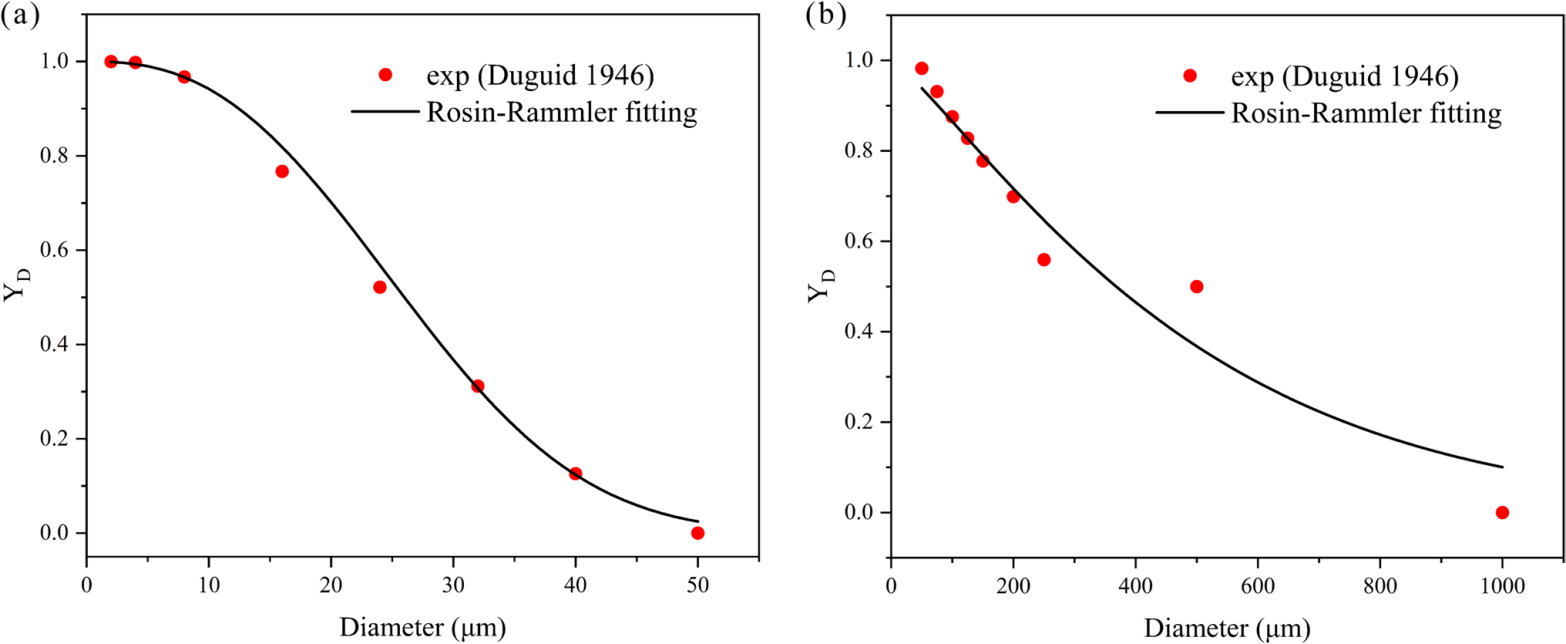
Rosin–Rammler distributions of cough droplet diameter. **a** 1 to 50 μm. **b** 50 to 1000 μm

The size of droplets expelled by coughing ranges from 1 to 1000 μm. Considering the specificity of the diameter distribution of large droplets and small droplets, we use two Rosin–Rammler distributions to fit the droplet diameter distributions of 1 to 50 μm and 50 to 1000 μm, respectively. In Rosin–Rammler distribution function, ${Y}_{d}$ is defined as the mass fraction of droplets with a diameter greater than $d$. And there is an exponential relationship between ${Y}_{d}$ and $d$ (Rosin and Rammler 1933):

6 $${Y}_{d}={e}^{{-(d/\overline{d})}^{n}}$$

where $\overline{d }$ is the mean diameter, and $n$ is the spread parameter, both of which are all obtained by fitting, as shown in Fig. 5. A total of 40,000 droplet parcels with a total mass of 7.7 mg are released evenly from 0.05 to 0.45 s through a cone injection with a diameter of 20 mm in front of the mouth (Zhu et al. 2006; Li et al. 2018). Each parcel represents a certain number of droplets according to the total mass and Rosin–Rammler distribution. The injection velocity of droplet parcels is 6.6 m/s (Nishimura et al. 2013), and the angle between the cough airflow and the horizontal is 24.7° (Tellier 2006) . The concentration of non-volatile components in cough droplets is 1.8% (Nicas et al. 2005), which means that the equilibrium diameter of the droplet nucleus is approximately 26% of its initial size.

We employ the discrete phase model (DPM) to analyze the trajectory of cough droplets. Based on Newton’s second law and considering the influence of drag force, gravity, and buoyancy, each droplet parcel is tracked for unsteady motion (Li et al. 2018):

7 $$\frac{d \vec{u}_{p}}{d t}=\frac{\vec{u}-\vec{u}_{p}}{\tau_{r}}+\frac{\bar{g}\left(\rho_{p}-\rho\right)}{\rho_{p}}$$

where ${\stackrel{\rightharpoonup }{u}}_{p}$ is the droplet velocity, ${\tau }_{r}$ is the droplet relaxation time, which depends on the diameter and density of the droplet, $\stackrel{\rightharpoonup }{g}$ is the gravity acceleration, and ${\rho }_{p}$ is the droplet density.

The random walk model is used to characterize the droplet dispersion caused by turbulence. The instantaneous turbulent velocity fluctuations are added to the local air velocity (Li et al. 2018):

8 $${u}_{i}=\overline{{u}_{i}}+\zeta \sqrt{\frac{2k}{3}}$$

where $\zeta$ is a normally distributed random number, and *k* is the turbulent kinetic energy.

Droplets evaporate gradually in the environment, and the nucleus diameter of a droplet is 26.2% of its initial diameter (Brzeźniak et al. 2008). The evaporation of droplets is controlled by the difference in water vapor concentration between the droplet surface and the environment (Li et al. 2018):

9 $$\frac{{dm}_{p}}{dt}=-\pi {d}_{p}{D}_{i,m}Sh\frac{{M}_{v}}{{M}_{m}}1\mathrm{n}\frac{1-{X}_{v,s}}{1-{X}_{v,m}}$$

where ${m}_{p}$ is the mass of droplets, ${d}_{p}$ is the diameter of droplets, ${D}_{i,m}$ is the diffusion coefficient; $Sh$ is the Sherwood number, which is the ratio of convective mass transfer to diffusion mass transfer, ${M}_{v}$ and ${M}_{m}$ are the molecular weights of the water vapor and the mixture, and ${X}_{v,s}$ and ${X}_{v,m}$ are the local mole fraction and equilibrium mole fraction of water vapor, respectively.

### Boundary conditions and numerical setup

Air change rates in high risk settings usually range from 6 to 12 air changes per hour (Jensen et al. 2005). Based on this recommendation, the fresh air is supplied through an air supply opening with an area of 0.48 m^2^ and a velocity of 2 m/s, corresponding to 12 air changes per hour. The supplied air temperature is 20 °C. The boundary condition of the exhaust vent is the pressure outlet. The temperature for clothes-surface of human body is 32 °C, which characterizes the heat flux transferred to the surrounding environment (Tao et al. 2017). Other solid walls are considered adiabatic. For the boundary conditions of the DPM model, the walls, ground, and ceiling are set to “trap,” meaning that droplets have been deposited after reaching the surface. The boundary condition of the exhaust vent and the air supply opening is set to “escape.”

Momentum, energy, *k*, and *ɛ* equations are all discretized by the second-order upwind scheme. The pressure term is discretized by the “PRESTO!” scheme to capture the pressure gradient at the boundary. And the couple algorithm is used to solve algebraic equations. Droplets are tracked within 100 s. In the cough airflow stage, the time step is set to 0.01 s to capture the cough jet airflow. After 10 s, the indoor flow hardly changes, and the time step is set to 0.1 s. The convergence criterion is that the energy relative residual reaches 10^−7^, the relative residuals of other variables reach 10^−4^, and convergence is reached in 10 to 20 iterations for each time step.

## Results and discussion

### Model validation

**Fig. 6:**
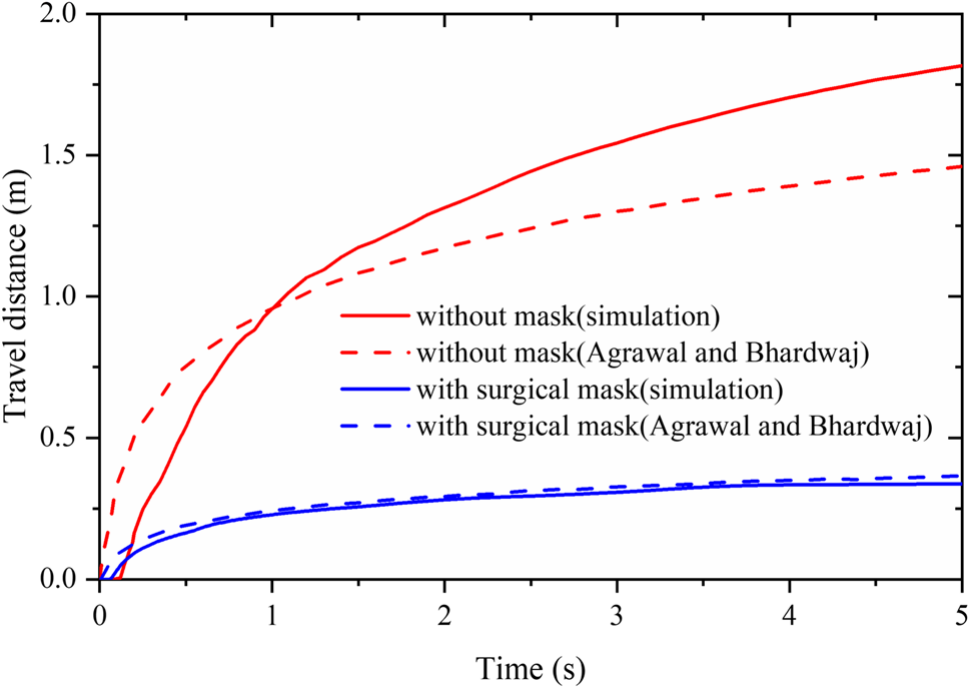
Movement distance of cough cloud front position over time

The DPM model has been widely verified and has good performance for predicting droplet movement and deposition (Lu et al. 1996). However, airflow has a significant impact on the movement of droplets. Before studying the lifetime of droplets expelled by coughing, we verify the accuracy of cough airflow phase. Agrawal and Bhardwaj established a mathematical model of the movement distance of cough cloud front in a closed space based on the experimental results (Agrawal and Bhardwaj 2020; Prasanna Simha and Mohan Rao 2020). In this validation study, transient CFD simulations are applied to two cases, namely, no face mask and surgical face mask. As the movement distance of cough cloud front is difficult to be monitored, we adopt the movement distance of particles with a diameter of 1 μm to evaluate the movement distance of cough cloud front. The particles are released in front of the mouth and follow the cough airflow closely. The result of movement distance of cough cloud front over time is shown in Fig. 6. At 5 s, the discrepancies of simulation and mathematical model are 7.7% and 24.8%, corresponding to cases with and without a face mask, respectively. The simulation results are slightly less than that of the mathematical model in the early stage of cough. And the discrepancy in the subsequent stages is relatively large for the case without a face mask, which may be caused by the difference in the initial velocity of the cough airflow between the simulation and the mathematical model. But the movement distance of cough cloud front is reasonable in the range of 1.5 to 3 m (Prasanna Simha and Mohan Rao 2020).

**Fig. 7:**
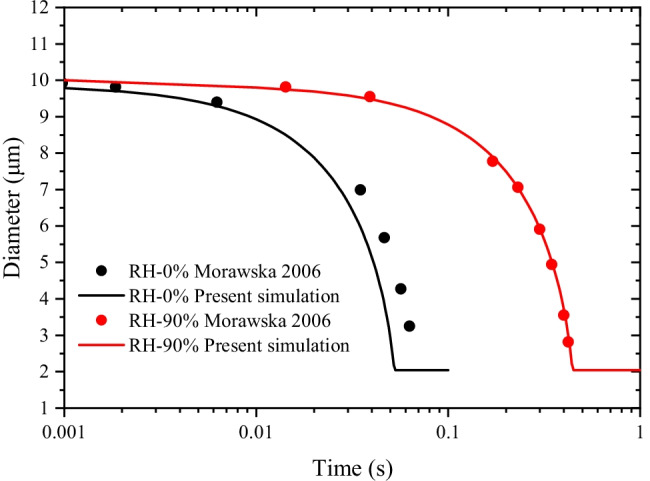
Validation of evaporation model

Moreover, the evaporation process of a droplet with a representative diameter of 10 μm in the enclosed chamber is compared with reported data (Morawska 2006). The temperature of the chamber is 25 °C, and the relative humidity is 0% and 90%, respectively. The simulation results are in good agreement with the literature (Fig. 7). The results also reveal that when the relative humidity of the chamber increases from 0 to 90%, the evaporation rate of 10 μm droplets reduces by nearly 10 times. The accurate prediction of droplet evaporation at two extreme cases of relative humidity validates the robustness of the simulation model.

### Cough dynamics

**Fig. 8:**
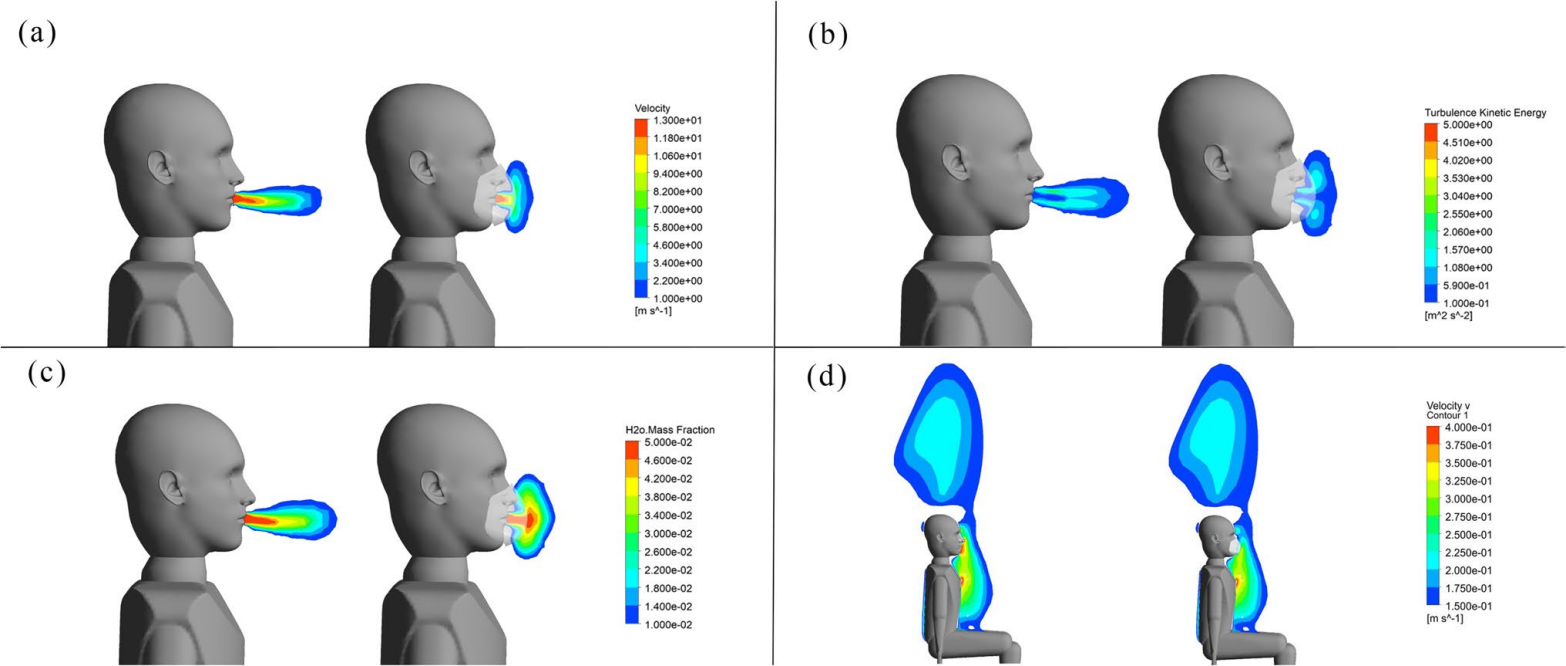
Flow characteristics in front of the individual. **a** The velocity magnitude at 0.1 s. **b** The turbulent kinetic energy at 0.1 s. **c** The mass fraction of water vapor at 0.1 s. **d** The velocity component in the *y*-direction at 10 s

Although the cough airflow dissipates quickly, the trajectories of droplets are greatly affected (Chen and Zhao 2010). We investigate the characteristics of airflow and droplets during a cough event when an individual wears a surgical face mask. Figure 8a–c show the velocity magnitude, turbulent kinetic energy, and mass fraction of water vapor at 0.1 s after a cough, respectively. Two small eddies are found between the face mask and the face compared to the condition without a face mask. As face mask limits the axial momentum, the velocity of exhaled airflow attenuates faster in this direction. At the same time, the water vapor accumulates around the face mask, resulting in greater relative humidity. The average mass fraction of water vapor in a spherical region in front of the individual is 1.78%. By contrast, it is 1.2% for the case without a face mask. We further investigate the turbulent kinetic energy and find that the maximum turbulent kinetic energy is about the same whether wearing a face mask or not at 0.1 s. But due to the resistance of face mask, turbulent kinetic energy is hard to diffuse between the face mask and the face. The fluctuating velocity of the cough jet increases, which may give rise to the leakage of droplets. At 10 s, the cough airflow is completely dissipated, and we observe that the thermal plume region around the individual is similar whether wearing a face mask or not (Fig. 8d).

**Fig. 9:**
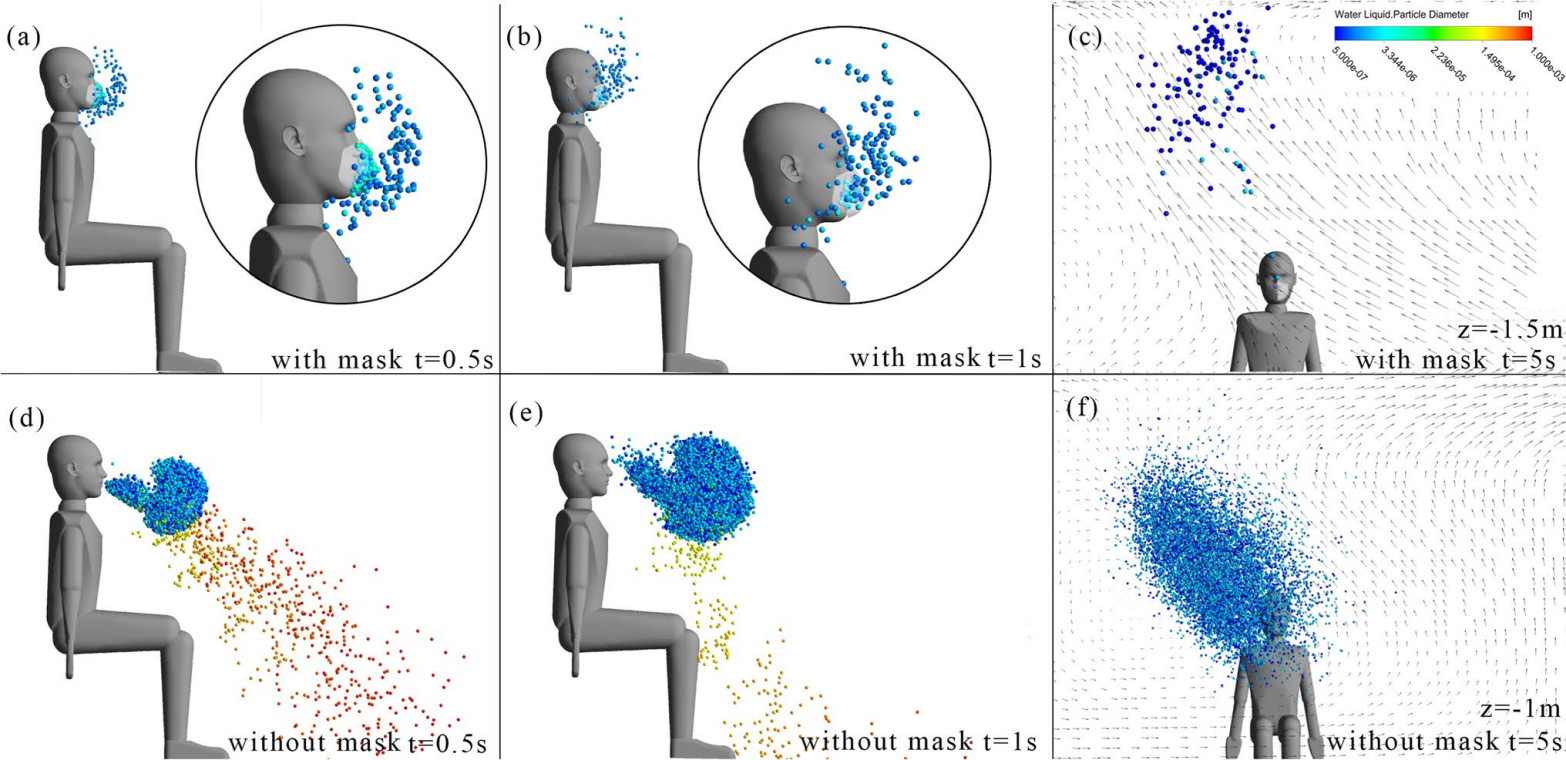
Droplets position at different moments with or without a face mask. **a** With mask at 0.5 s. **b** With mask at 1 s. **c** With mask at 5 s. **d** Without mask at 0.5 s. **e** Without mask at 1 s. **f** Without mask at 5 s

Qualitative comparison of droplet distribution with a face mask for a short period after cough is performed (Fig. 9). In the early period of cough, droplets with a diameter greater than the cutoff diameter of the face mask (4 μm) hit the surface of face mask and are stuck. Other droplets tend to follow the airflow due to the small relaxation time. As a result, most of the droplets with a diameter less than 4 μm are also stuck by a face mask, and a small part of the droplets pass through the face mask. At the later stage, some droplets leak through the gap between the face mask and the face. At 5 s, residual droplets are mainly distributed in front of the individual about 0.2 m since the face mask resists the momentum of exhaled airflow. However, without a face mask, most of the droplets are distributed in a zone about 0.7 m in front of the individual and completely dominated by the main flow stream. In summary, with a face mask, the quantity of droplets is significantly reduced and the residual ones are closer to the individual after a short period of cough.

### Quantitative analysis

**Fig. 10:**
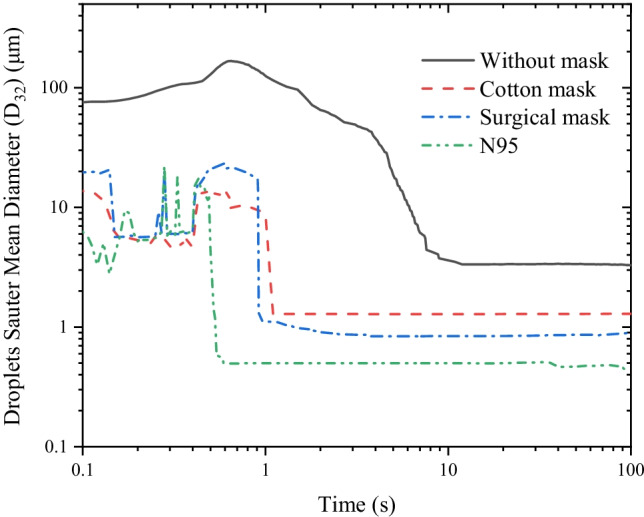
Sauter mean diameter of cough droplets in the room over time

A more detailed quantitative investigation on the droplet lifetimes is carried out when an individual wears different types of face masks. Due to the influence of droplet evaporation, sedimentation, and sticking on the face mask surface, the diameter distribution of droplets varies constantly in the room. The diameter of droplets directly affects their transmission behavior. Sauter mean diameter (SMD), also known as *D*_32_, is implemented to quantify the evolution of droplet size over time (Fig. 10):

10 $${D}_{32}=\frac{{\int }_{{D}_{min}}^{{D}_{max}}{D}^{3}dN}{{\int }_{{D}_{min}}^{{D}_{max}}{D}^{2}dN}$$

where $D$ is the droplet diameter, and $N$ is the quantity of droplets with the diameter $D$.

We find that *D*_32_ is about 93.2 μm within the period of the first 0.5 s without a face mask. From 0.5 to 10 s, *D*_32_ decreases from 128.9 to 3.6 μm due to the evaporation and sedimentation of droplets. From 10 to 100 s, it is almost stable around 3.3 μm. By wearing a face mask, *D*_32_ drops rapidly within 1 s because of the sticking of droplets on the face mask surface. Subsequently, the remaining droplets are exposed to a room by passing through the face mask or leaking through the gap. And the *D*_32_ drops slightly to 1.3 μm, 0.90 μm, and 0.45 μm at 100 s due to evaporation, corresponding to wearing cotton face masks, surgical face masks, and N95 face masks, respectively. The difference in *D*_32_ is mainly attributed to the difference in the cutoff diameter of face masks. These small droplets may suspend for several hours or even not settle at all (Tellier 2006), which causes the indoor exposure risk.

**Fig. 11:**
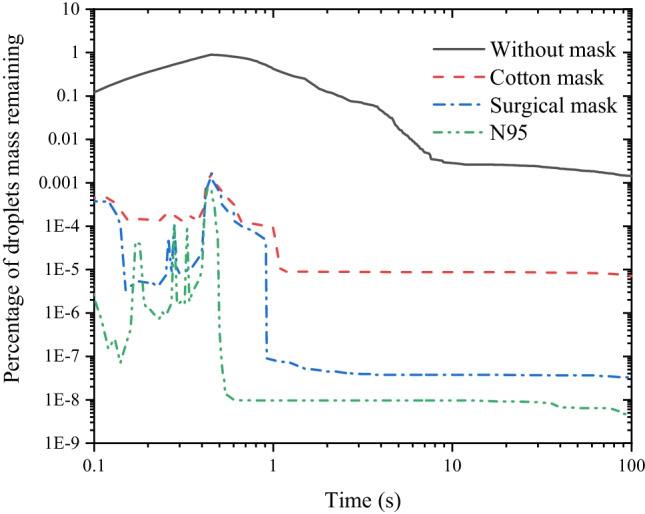
The percentage of residual droplet mass over time

The residual droplet mass can be used to characterize the virus concentration in the room. We further examine the ratio of residual droplet mass to inject droplet mass over time (Fig. 11). With a face mask, the percentage of residual droplet mass rises to about 0.1% with the continuous release of droplets and accumulation between the face and the face mask during the initial 0.5 s. After that, its value declines rapidly because the relatively large diameter droplets with a larger mass are stuck by the face mask. However, the maximum value of the percentage of residual droplet mass is about 89.3% without a face mask. At 10 s, the residual mass of droplets in the room accounts for 0.29% of the inject droplets mass without a face mask. By contrast, the value is only $8.7\times {10}^{-6}$, $3.7\times {10}^{-8}$, and $9.7\times {10}^{-9}$ corresponding to wearing cotton face masks, surgical face masks, and N95 face masks, respectively. After that, the residual mass of droplets decreases gradually due to the influence of the ventilation system and droplet sedimentation. In general, the use of face masks reduces the concentration of virus-carrying droplets in the room by 2 to 6 orders of magnitude. In other words, even a simple cotton face mask can effectively prevent the transmission of the virus.

**Fig. 12:**
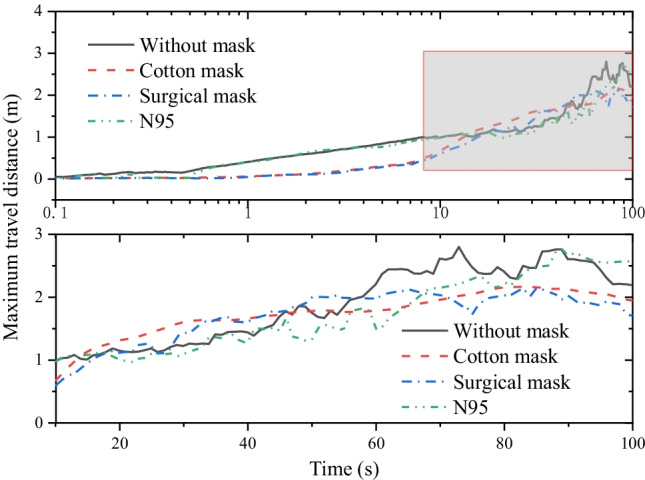
The maximum travel distance of 80% droplets over time

To evaluate the influence range of cough droplets, we introduce a parameter, the maximum travel distance of 80% droplets in a room, which we shall name metric distance for short. As shown in Fig. 12, the metric distance with a face mask is significantly shorter than that of not wearing a face mask in the first 10 s. This is mainly attributed to the resistance of the face mask to the exhaled airflow and the sticking of the high-momentum droplets. But for a long time, the remaining droplets join the main flow stream and are dominated by it. Therefore, the metric distance is similar with or without a face mask, which is approximately reaching about 2 m in 100 s. Despite wearing a face mask, there are still a small number of residual droplets traveling a long distance. The ultraviolet germicidal irradiation system can be considered to reduce this risk due to its ability to inactivate microorganisms (Hope and Harrington 2020).

### Aerosol exposure risks

**Fig. 13:**
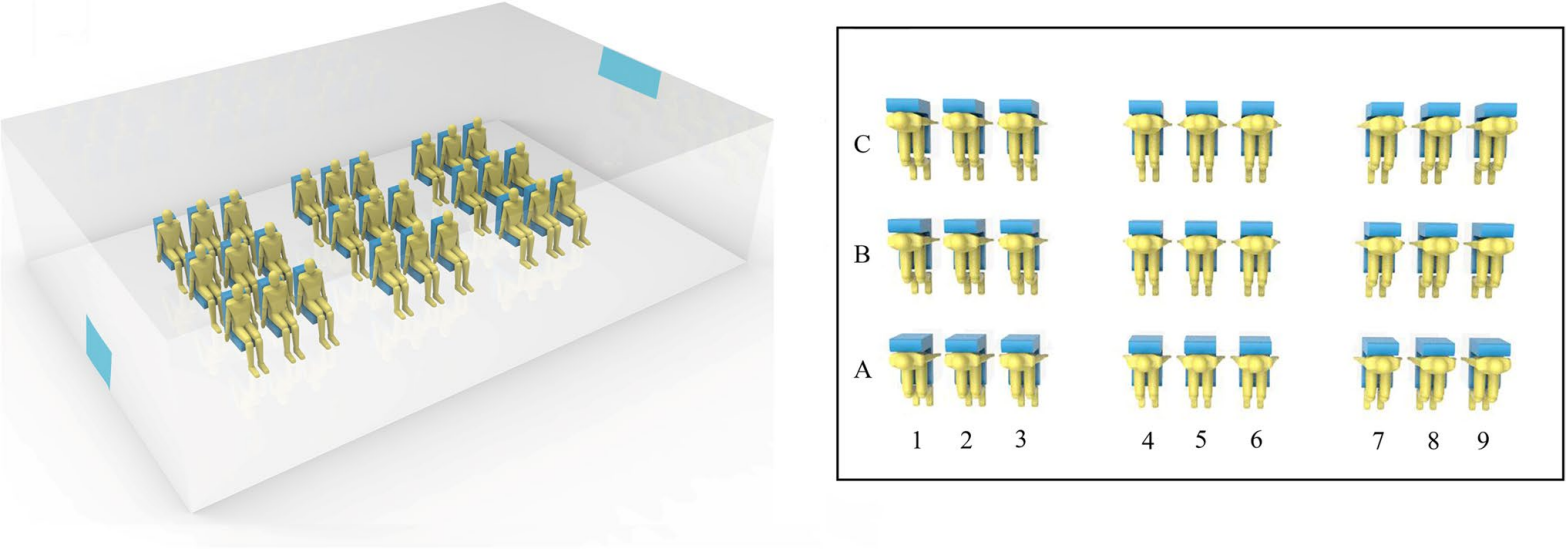
Schematic diagram of a ventilated room with 27 individuals

Through the above analysis, we realize that the main risk in a room is aerosol exposure even when a sick individual wears a face mask. Suppose there are 27 individuals sitting in a room, and there is only one infected individual. As shown in Fig. 13, the infected individual continuously releases aerosols with a diameter of 2 μm at several representative locations, which can represent the transmission behavior of the vast majority of aerosols under the condition of indoor environment. The fresh air is supplied through an air supply opening on the top right of the diagram. It is assumed that the infected individual wears a surgical face mask and the exhaled airflow velocity is 5 m/s. The particle source in cell method is used to convert the aerosol trajectory into a concentration field, as expressed in Eq. (11) (Zhang and Chen 2007):

11 $${C}_{j}=\frac{M\sum_{i=1}^{m}dt(i,j)}{{V}_{j}}$$

where ${C}_{j}$ is the aerosol concentration in the cell, $M$ is the mass flow through the cell, $dt\left(i,j\right)$ is the residence time of the aerosol in the cell, and ${V}_{j}$ is the mesh volume. A large number of studies have proved the reliability and suitability of this method (Wang and Chow 2011; Zhang and Li 2011; Yan et al. 2017).

**Fig. 14:**
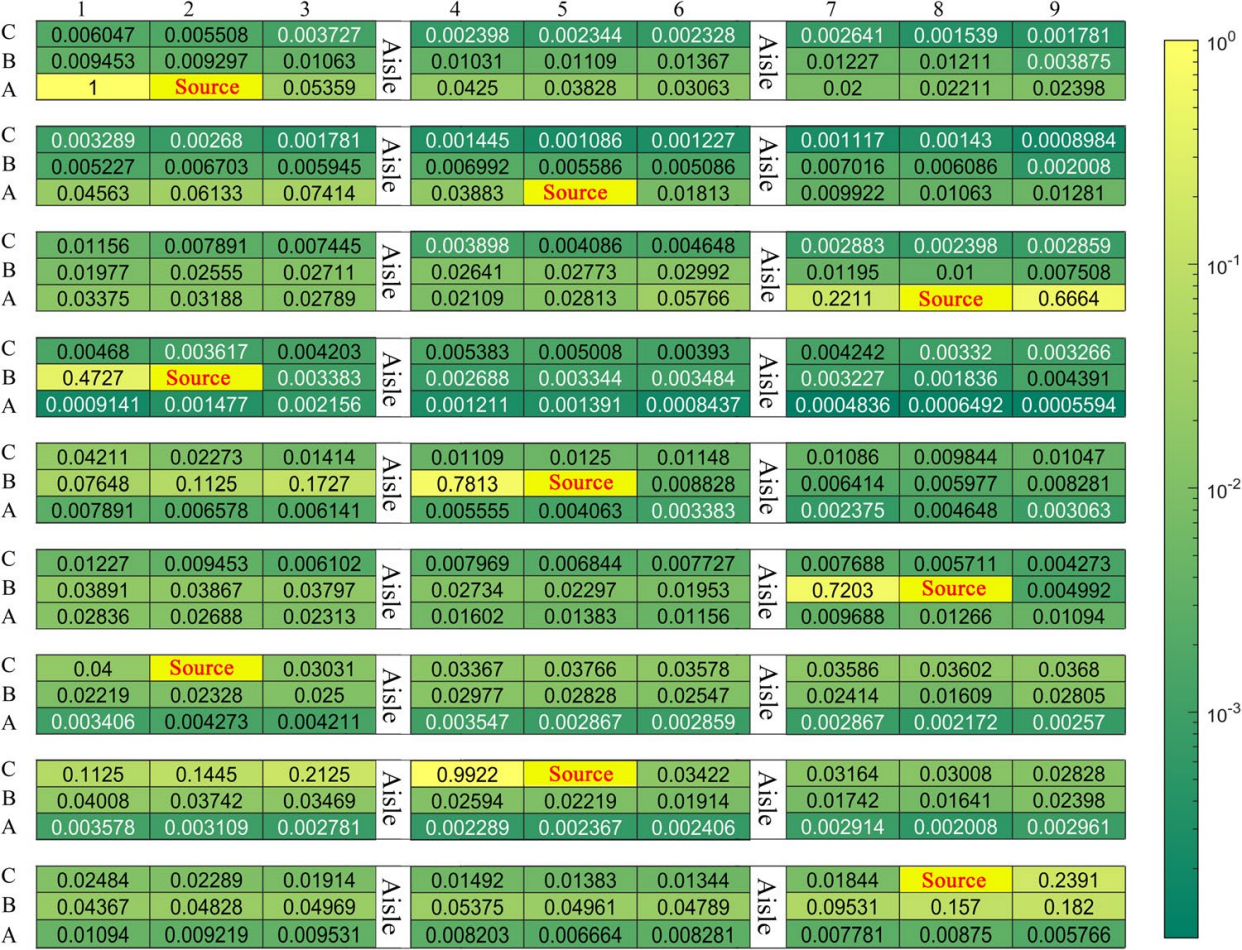
Heatmap of the aerosol normalization concentration. The distributions of aerosol normalization concentration in breathing zones for the infected individual (source) at 2A, 5A, 8A, 2B, 5B, 8B, 2C, 5C, and 8C, respectively

A spherical area with a radius of 0.3 m in front of each individual is defined as the breathing zone (Yan et al. 2017). The aerosol concentration in the breathing zone is volume-averaged and normalized. We consider 9 cases, in which the infected individual is located in A2, A5, A8, B2, B5, B8, C2, C5, and C8, respectively. The distributions of aerosol normalization concentration are summarized in Fig. 14. Individuals in row B and the same row of the infected individual have an increased risk of infection when infected individual releases aerosols at different locations, while aerosols hardly transmit to the other half of the area due to the existence of a couple of large re-circulation. A couple of large re-circulations have opposite directions and split the airflow into two areas. Moreover, when the infected individual is close to the air supply opening (i.e., column 5, column 8), other individuals in the same row are at higher risk of infection, especially individuals on the left side of the infected individual. However, when the infected individual is near the exhaust vent (column 2), aerosols are quickly taken away by the injected airflow, and individuals in other locations are relatively safe.

**Fig. 15:**
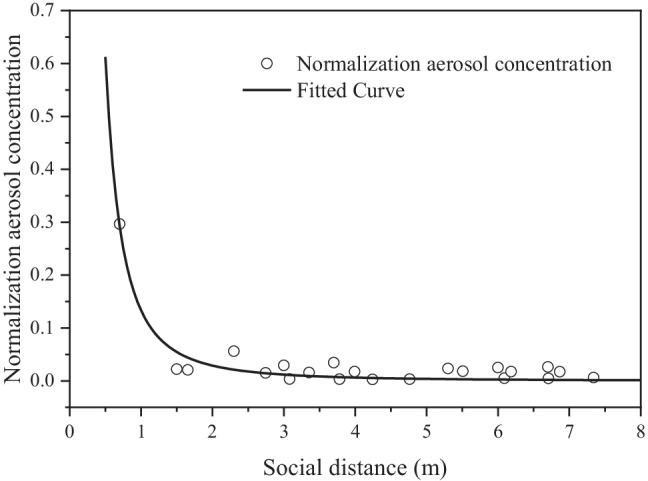
Aerosol normalization concentration over social distance

To stop the transmission of the virus, social distancing has been widely applied in most countries (Srivastava et al. 2020). To evaluate the effectiveness of social distancing for indoor activities, the trend of normalized concentrations of aerosols in the above 9 cases over social distance is provided (Fig. 15). When the distance from an individual with a face mask is smaller than 1.5 m, a high concentration is observed. By contrast, when the distance is greater than 1.5 m, the concentration remains at a low level. Therefore, a minimal distance of 1.5 m is required to reduce the spread of the virus for indoor activities, such as lectures, conferences, and office work, together with wearing face masks.

## Conclusions and implications

In this study, the transmission characteristics of droplets expelled by a single cough with and without a face mask in a ventilated room are studied by numerical simulations. We consider a face mask as a porous medium and examine its effects on the airflow and droplets expelled by a relatively complex human body model. The simulation results demonstrate that in the early stage of a cough event, the droplet travel distance is shorter with wearing a face mask, and the quantity of droplets in a room is much smaller compared to the condition without a face mask. A long time after cough, by wearing a face mask, even a cotton face mask, the *D*_32_ and residual mass of droplets in a room are much lower than that without a face mask. The concentration of virus-carrying droplets in the room decreases by 201, 43,786, and 307,060 times, corresponding to wearing cotton face masks, surgical face masks, and N95 face masks, respectively. But the maximum travel distance of 80% droplets is similar in both cases. We further investigate aerosol exposure risks in different areas of the room and find that although an infected individual faces forward, high concentrations of aerosols occur in the streamline through the infected individual, especially next to the individual within 1.5 m.

This study indicates that the use of face masks can avoid a large quantity of cough droplets to be released into the indoor environment, which greatly reduces the risk of infection for healthy people in the surrounding area. Therefore, it is strongly recommended that people wear face masks in public. However, it should be noted that droplets passing through face masks or leaking through the gap between the face mask and the face are still widely distributed in the room and difficult to settle due to the influence of indoor airflow. Considering that SARS-CoV-2 may be transmitted through aerosols, these small droplets suspended indoors should not be ignored. The 1.5 m social distance should still be maintained despite wearing face masks, regardless of the positional relationship between individuals. Moreover, the ultraviolet germicidal irradiation system can be considered to reduce indoor infection risks due to its ability to inactivate microorganisms. From the perspective of indoor airflow, no individuals should be sitting near the air supply opening to prevent cough droplets from spreading widely in the room. In addition, personalized ventilation and installing partitions to separate individuals could be considered to reduce the risk of cross-infection. In comparison to other studies, this study has reported more detailed dynamic information related to the transmission of droplets during a cough with or without a face mask in a public ventilated room. Although this research is under ideal conditions, its findings help policy makers understand the protective mechanism of face masks to effectively execute corresponding measures for public health.

Finally, to simplify the simulation, this study ignores the secondary breakup of cough droplets and the rebound of droplets when contacting walls and individual surfaces. The influence of these factors needs to be explored in future work. In addition, it is also necessary to improve the understanding of the following aspects, including the mechanism of droplet penetration through face masks, the impact of the gap between the face mask and the face on the protective effect, and the quantitative relationship between droplet mass and virus concentration. Further research needs to be carried out to better tackle the challenge of SARS-CoV-2 transmission.

## Data Availability

The datasets used and/or analyzed during the current study are available from the corresponding author on reasonable request.
